# Measuring the Complex Optical Conductivity of Graphene by Fabry-Pérot Reflectance Spectroscopy

**DOI:** 10.1038/srep34166

**Published:** 2016-09-29

**Authors:** Behnood G. Ghamsari, Jacob Tosado, Mahito Yamamoto, Michael S. Fuhrer, Steven M. Anlage

**Affiliations:** 1 Center for Nanophysics and Advanced Materials, Department of Physics, University of Maryland, College Park, MD, 20742-4111, USA

## Abstract

We have experimentally studied the dispersion of optical conductivity in few-layer graphene through reflection spectroscopy at visible wavelengths. A laser scanning microscope (LSM) with a supercontinuum laser source measured the frequency dependence of the reflectance of exfoliated graphene flakes, including monolayer, bilayer and trilayer graphene, loaded on a Si/SiO_2_ Fabry-Pérot resonator in the 545–700 nm range. The complex refractive index of few-layer graphene, *n* − *ik*, was extracted from the reflectance contrast to the bare substrate. It was found that each few-layer graphene possesses a unique dispersionless optical index. This feature indicates that the optical conductivity does not simply scale with the number of layers, and that inter-layer electrodynamics are significant at visible energies.

Optical response of graphene has recently emerged as an important subject of research. This interest is partly fueled by the promising prospect of utilizing graphene as a photonic material for device applications^1^,^2^,^3^,^4^,^5^,^6^,^7^. Nevertheless, optical response can probe numerous important properties of graphene such as the high-frequency screening, optical conductivity, and many-body effects. It is well-established that the low-energy spectrum of quasiparticles in graphene is governed by a linear energy-momentum relation. In fact, a plethora of transport, quantum Hall, and high-frequency experiments have provided ample evidence for the band structure linearity from DC to terahertz frequencies^8^. A remarkable consequence of a linear energy-momentum dispersion in two dimensions is a universal optical conductivity^9^,^10^,^11^. Therefore, testing the universality of graphene’s optical conductivity can assess the applicability of the Dirac fermion picture and the extent of many-body effects at higher energies.

The observation of highly confined and low-loss plasmons at mid-infrared wavelengths^12^,^13^ has demonstrated that the universality sustains at least up to infrared (IR) energies. Extending the experiments to higher frequencies, however, has proven challenging. In order to achieve low damping, the plasmon frequency should lie between the optical phonon threshold and the onset of the interband transition^14^,^15^,^16^. Otherwise, plasmon oscillations are drastically suppressed by scattering from optical phonons or Landau damping, respectively. The required Fermi level for translating the onset of the interband transitions to near-IR/visible energies could not be readily obtained by electrostatic or chemical doping (~10^15^ *cm*^−2^).

On the other hand, the observation of saddle-point excitons^17^ has demonstrated that many-body effects significantly factor in at ultra-violet (UV) energies. Given that the universality of the optical conductivity is well supported by a number of methods up to infrared (IR) frequencies, it has yet remained ambiguous as to whether the *low-energy* approximation breaks down, and the linear band structure and a universal optical conductivity are violated. Presuming that transition from a linear energy dispersion (at IR) to a non-linear one (at UV) occurs smoothly, it is plausible that tangible deviations from the former should begin to appear in the visible regime.

In this regime, the optical response of thin films may be probed by several methods including near-field microscopy, ellipsometry, and transmission/reflection measurements. The use of near-field probes for interrogating visible graphene plasmons is limited due to the interband transitions and high dissipation under conventional doping levels, as highlighted earlier. Moreover, ellipsometry techniques normally suffer from a low spatial resolution, hindering measurement of small graphene flakes. Therefore, transmission/reflection experiments have constituted the most widely used means to characterize few-layer graphene in the visible spectrum.

Early optical reflection measurements^18^,^19^,^20^,^21^,^22^,^23^,^24^,^25^ on graphene were mainly focused on resolving the number of graphene layers and, most significantly, identifying monolayer graphene among other flakes. Therein, the dependence of the reflectance on the flakes’ thickness were exploited to count the number of graphene layers. Quantitative models associated with these experiments, in large part, involved assuming a constant refractive index over the entire visible range common to all graphene multi-layers. The value of this dispersionless optical index ought to be extracted, based on the Fresnel reflection theory^26^, from the measured contrast between the reflectance of the supported graphene flake and that of the bare substrate. A dispersionless optical index, however, contradicts a universal optical conductivity from the onset since from Ampere’s law 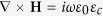

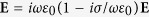
. Here, **H** and **E** are the magnetic and electric fields, respectively, *ε*_0_ = (n–ik)^2^ is the permittivity of free space, *ε*_*c*_ is the complex relative permittivity, σ is the optical conductivity, and *ω* is the angular frequency of the incident monochromatic optical field. Clearly, if the refractive index, and, consequently, dielectric function *ε*_*c*_ are independent of frequency, then *σ* must necessarily be a linear function of *ω*. Although the precise complex value of the deduced refractive index varies among different experimental determinations, they are closely clustered within a small range: 2.1 < n < 2.7 and 1.0 < k < 1.7.

Nonetheless, the best-known transmission measurement was performed on suspended few-layer graphene^27^. Therein, it was observed that the transmittance varied in steps of ≈2.3% with the number of layers for white light. The scaling of opacity with the number of layers was interpreted as the predominance of the in-plane electrodynamics, compared to interlayer dynamics, in few-layer graphene. More importantly, the experiment showed that the 2.3% reduction in the transmittance for monolayer graphene held over almost the entire visible range. This result has been widely regarded as a proof that the universality of the optical conductivity sustains even at optical energies. Close examination of the measured spectrum, however, reveals noticeable deviations from a universal optical conductivity for wavelengths below 500 nm. Although this point was recognized by the authors, hydrocarbon contamination was suggested as its origin. Interestingly, other groups have also observed departure from a universal optical conductivity in the same energy scale^17^,^28^,^29^. The deviation well fits, to first approximation, to a linear increase in the optical conductivity versus energy^29^. For example, the linear approximation to the optical conductivity in the 1–2 eV range may be inferred, for instance, from Fig. 1 in ref. 17. This is consistent with the dispersionless optical index assumption which was adopted in the early works. Furthermore, as will be discussed later on, both the 2.3%-opacity-steps as well as a nearly constant opacity over the visible range may be well obtained from a properly chosen value for a dispersionless optical index. Therefore, whether the universal optical conductivity hypothesis holds up to visible frequencies still remains unresolved.

This paper reexamines the optical response of few-layer graphene over the visible range by means of a refined reflectance spectroscopy technique. In particular, we compare the two hypotheses of universal optical conductivity and dispersionless optical index. Note that the latter implies a dispersive optical conductivity varying linearly with frequency.

Since the thickness of graphene is several orders of magnitude smaller than an optical wavelength, transmission through graphene essentially samples only the real part of the optical conductivity. The imaginary part, however, manifests in the reflection. The negligible amplitude of the reflection from suspended graphene, thus, renders transmission measurements insensitive to the imaginary part. The same disadvantage persists for reflection measurements where graphene films are directly laid on a substrate. Therein, the reflected power predominantly originates from the reflection at the substrate interface. In order for both the real and imaginary parts of the optical conductivity/index to affect the measured signal, we have coupled the graphene flakes to a Fabry-Pérot resonator. The Fabry-Pérot cavity may be simply realized by deposition of a thin dielectric film on the substrate, whose thickness determines the resonant frequency. Given that the overall reflectance from a Fabry-Pérot structure results from multiple reflections, the effect of the interaction with graphene is magnified. While the phase retardation induced by graphene may be negligible for a single reflection, its accumulation for many reflections, however, yields a measurable effect. This fact has been previously exploited to identify different few-layer graphene flakes^19^.

## Experiment

**Figure 1:**
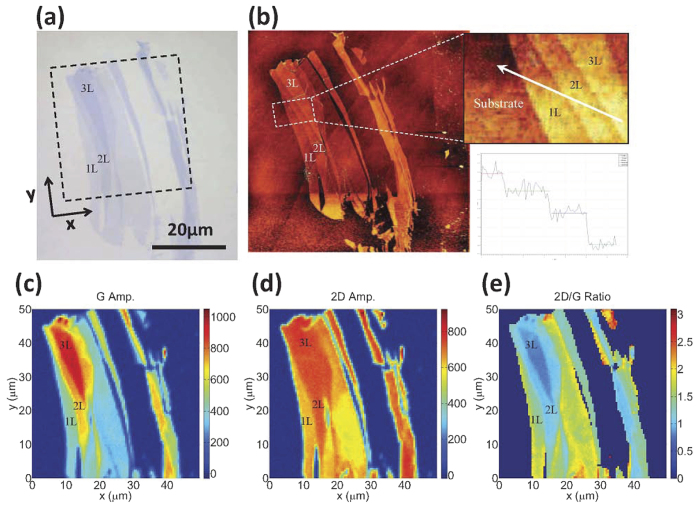
(**a**) Optical image of the exfoliated flake. The black square marks the region studied in the subsequent reflectance measurements. (**b**) AFM images of the flake with a line-cut showing single graphene layer steps. (**c–e**) Amplitudes of the G- and 2D- peaks in the Raman spectrum as a function of position and their ratio, respectively.

A graphene flake was exfoliated onto a commercially available Si substrate with a nominally 300 nm-thick SiO_2_ over-layer. This particular SiO_2_ thickness provides a third-order Fabry-Pérot absorption resonance corresponding to an antinode at the graphene layer around a (free-space) wavelength of 550 nm. The sample was then annealed in H_2_ and Ar at a temperature of 500 °C for 3 hours to remove adsorbed contaminants. Figure 1a shows a broadband optical image of the graphene flake.

Regions of mono-layer, bi-layer, and tri-layer graphene were identified by means of Raman spectroscopy^30^ and atomic force microscopy (AFM), Fig. 1b, where the latter was performed after the reflectance measurements to prevent possible damage to the surface. Mapping the regions of different few-layer graphene were complemented by means of the spatially resolved amplitudes of the G and 2D Raman peaks, as Fig. 1c,d depict. Figure 1e, moreover, shows that the ratio of the amplitude of the 2D peak to that of the G peak monotonically decreases with increasing number of layers and is, in fact, an efficient tool in identifying the thickness of the flakes. However, it should be noted that, due to the finite spatial resolution of the Raman microscope, the ratio of the two peaks may give rise to spuriously high values near the edges. This effect is more pronounced near the graphene-substrate boundary where a rapid decline in the amplitude of the G peak on the substrate might result in a false large ratio.

**Figure 2:**
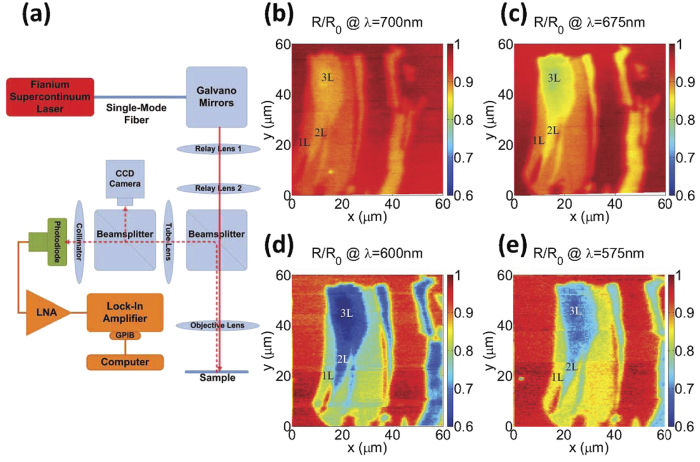
(**a**) Schematic of the measuring LSM apparatus. (**b–e**) Measured reflected light from the sample surface at an incident wavelength of 700 nm, 675 nm, 600 nm, and 575 nm, respectively. The images are normalized to the reflected light from the bare substrate (*R*_0_) at the corresponding wavelengths in order to manifest variations of the visibility of graphene with wavelength.

Reflectance measurements were performed by a laser scanning microscope (LSM)^31^, as illustrated in Fig. 2a, which raster-scans a focused laser spot of nominally 1 *μm* diameter over the sample using a set of two galvano-mirrors. An X20 Mitutoyo infinity-corrected long working-distance objective lens, with a NA of 0.42, was used to focus the incident light onto and gather the reflected light from the sample. Given the flake dimensions (less than 60 *μ*m on a side) and the working distnace of 2 mm, the deviation of the incidence angle from normal is less than 0.9°. The reflected beam was directed to a Si photodiode which measured the reflected power at each pixel.

A Fianium SC400 supercontinuum laser was used as the LSM light source whose wavelength was varied from 545 nm to 700 nm by means of an acousto-optic tunable filter with a bandwidth of <2 nm.

The thickness of the oxide layer enters to the data analysis (below) as an input parameter and its accurate determination is essential to the accuracy of the ultimate results. Therefore, despite the fact that ellipsometry and scanning electron microscopy (SEM) were performed on the substrate, the actual thickness of the SiO_2_ near the graphene flake was determined, after the reflectance measurements, through selectively removing the oxide by hydrofluoric acid etching followed by AFM, which gave a value of 308 nm ± 0.5 nm for the oxide thickness adjacent to the exfoliated graphene flake.

## Results and Discussion

As Fig. 2b–e illustrate, the contrast of different few-layer graphene to the bare substrate varies with the incident wavelength. These images, furthermore, show that the contrast to the substrate as well as among the graphene few-layers change at different rates. For example, graphene bi-layer and tri-layer become hardly distinguishable at a wavelength of 575 nm and 700 nm, as shown in Fig. 2b,e, whereas they could be easily identified at 600 nm and 675 nm, as shown in Fig. 2c,d. The graphene monolayer, however, keeps its contrast with the thicker flakes for the entire range. These observations imply that each few-layer may possess its own unique dielectric function.

**Figure 3:**
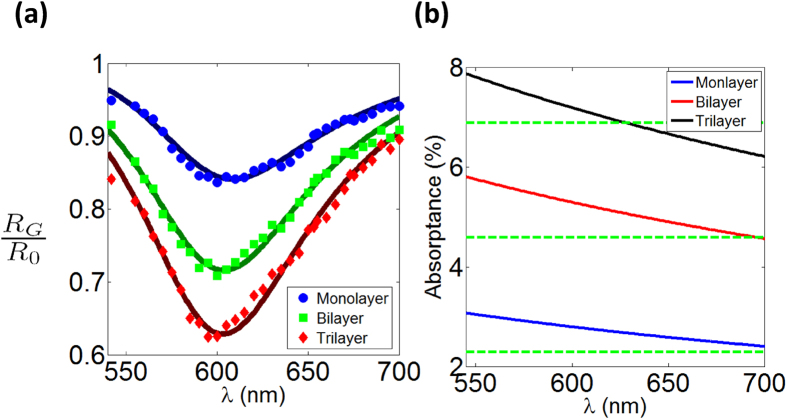
(**a**) Normalized reflectance of different few-layer graphene regions as a function of wavelength. Normalized reflectance is defined as the ratio of the graphene reflectance to that of the bare substrate at the same wavelength. Circles, squares, and diamonds respectively show the experimental data for monolayer, bilayer, and trilayer graphene and the solid lines are the best fit based on the Fresnel theory for each individual graphene layer irrespective of the others. (**b**) Opacity of suspended few-layer graphene as a function of wavelength calculated based on the refractive indices obtained in Table 1. Green dashed-lines show the previously reported 2.3%-opacity-per-layer for suspended graphene^27^.

Figure 3a shows the frequency-dependent reflectance for different graphene few-layers, *R*_*G*_, normalized by that of the bare substrate, *R*_0_. The measured reflectance for each graphene few-layer followed a Fabry-Pérot lineshape centered around ~600 nm. The contrast to the substrate monotonically increased for thicker graphene few-layers. This is qualitatively anticipated since thicker flakes absorb more and appear darker. Interestingly, the reflectance spectra underwent a slight red shift for thicker flakes. The resonance shift evidently demonstrates the loading effect of graphene on the Fabry-Pérot cavity. This shift is a manifestation of the significance of the imaginary (real) part of the optical conductivity (index).

In the literature, reflection/transmission analyses on graphene usually involved applying thin-film approximations, which neglect the phase retardation associated with transmission through and reflection from graphene. Here we used a complete Fresnel analysis on the graphene-loaded Fabry-Pérot multilayer cavity. The substrate was modeled as a semi-infinite medium. A 3.3 Å thickness was assumed for each layer of graphene, corresponding to its van der Waals interlayer distance. The refractive indices of Si and SiO_2_ were obtained from ref. 32 including their normal dispersion.

The dielectric functions of graphene were modeled based on: first a universal optical conductivity and, second, a complex constant over the wavelength range of interest. These two forms were incorporated into the Fresnel theory in order to explain the measured data, as shown in Fig. 3a. We found out that the dielectric functions based on a universal optical conductivity failed to reproduce the measured reflectance spectra, our first main conclusion. On the other hand, those based on a dispersionless optical index resulted in very good agreement with the measured data. The solid curves in Fig. 3a represent the calculated reflectance spectra and Table 1 lists the deduced optical indices.

The deduced optical refractive indices were, further, used in order to calculate the opacity of suspended few-layer graphene based on the Fresnel theory. Figure 3b displays the calculated absorptance as a function of the wavelength over the range where the experiments were conducted. The horizontal dashed lines represent the universal optical conductivity predictions, namely 2.3% per layer. The results of both models are close; especially for white light a dispersionless optical index model gives an average absorptance approaching to 2.3% ± 0.4% per layer.

The fact that each graphene few-layer possesses a different optical index is also interesting. It has been previously argued that the equal steps in the opacity of few-layer graphene indicate that the electrodynamics of graphene is predominantly determined by its in-plane response. However, the result that each few-layer graphene exhibits a unique optical index suggests that inter-layer electrodynamics significantly affect the optical response of graphene in the visible regime. Moreover, the difference in the imaginary parts imply that optical conductivity does not simply scale with the number of layers.

## Conclusions

We performed reflectance spectroscopy on exfoliated monolayer, bilayer, and trilayer graphene coupled to a Fabry-Pérot resonator by means of a laser scanning microscope and a supercontinuum laser over the wavelengths of 545 nm to 700 nm, corresponding to photons of energy 1.77 eV to 2.28 eV. The optical index of the few-layer graphene flakes were determined based on their contrast to the bare Si-SiO_2_ substrate by applying the Fresnel theory of reflection/refraction to the air/graphene/SiO_2_/Si multilayer. We found that the reflectance spectra can be well explained by a dispersionless effective optical refractive index rather than a constant optical conductivity. Furthermore, it was found that each few-layer possesses a unique optical index, as tabulated in Table 1. This, in turn, implies that the optical conductivity of graphene does not scale with the number of layers and inter-plane dynamics play a significant role in the graphene optical response in the visible regime.

## Additional Information

**How to cite this article**: Ghamsari, B. G. *et al*. Measuring the Complex Optical Conductivity of Graphene by Fabry-Pérot Reflectance Spectroscopy. *Sci. Rep.* **6**, 34166; doi: 10.1038/srep34166 (2016).

## Figures and Tables

**Table 1:** Dispersionless optical index (*n* − *ik*) of graphene obtained from the reflectivity spectroscopy.

Graphene	n	k
Monolayer	2.69 ± 0.02	1.52 ± 0.02
Bilayer	2.38 ± 0.02	1.66 ± 0.02
Trilayer	2.27 ± 0.02	1.60 ± 0.02
