# Erratum: Measuring the Complex Optical Conductivity of Graphene by Fabry-Pérot Reflectance Spectroscopy

**DOI:** 10.1038/srep40973

**Published:** 2017-01-19

**Authors:** Behnood G. Ghamsari, Jacob Tosado, Mahito Yamamoto, Michael S. Fuhrer, Steven M. Anlage

Scientific Reports 6: Article number: 34166; 10.1038/srep34166 published online: 09 29 2016; updated: 01 19 2017.

This Article contains an error in the Introduction section.

“Here, **H** and **E** are the magnetic and electric fields, respectively, *ε*_0_ = (n − ik)^2^ is the permittivity of free space, *ε*_*c*_ is the complex relative permittivity, σ is the optical conductivity, and *ω* is the angular frequency of the incident monochromatic optical field.”

should read:

“Here, **H** and **E** are the magnetic and electric fields, respectively, *ε*_0_ is the permittivity of free space, *ε*_*c*_ = (n − ik)^2^ is the complex relative permittivity, σ is the optical conductivity, and *ω* is the angular frequency of the incident monochromatic optical field.”

